# Spatio-Temporal Progression of Cortical Activity Related to Continuous Overt and Covert Speech Production in a Reading Task

**DOI:** 10.1371/journal.pone.0166872

**Published:** 2016-11-22

**Authors:** Jonathan S. Brumberg, Dean J. Krusienski, Shreya Chakrabarti, Aysegul Gunduz, Peter Brunner, Anthony L. Ritaccio, Gerwin Schalk

**Affiliations:** 1 Department of Speech-Language-Hearing: Sciences & Disorders, University of Kansas, Lawrence, KS, United States of America; 2 Department of Electrical & Computer Engineering, Old Dominion University, Norfolk, VA, United States of America; 3 J. Crayton Pruitt Family Dept. of Biomedical Engineering, University of Florida, Gainesville, FL, United States of America; 4 National Center for Adaptive Neurotechnologies, Wadsworth Center, New York State Department of Health, Albany, NY, United States of America; 5 Department of Neurology, Albany Medical College, Albany, NY, United States of America; Universitat Zurich, SWITZERLAND

## Abstract

How the human brain plans, executes, and monitors continuous and fluent speech has remained largely elusive. For example, previous research has defined the cortical locations most important for different aspects of speech function, but has not yet yielded a definition of the temporal progression of involvement of those locations as speech progresses either overtly or covertly. In this paper, we uncovered the spatio-temporal evolution of neuronal population-level activity related to continuous overt speech, and identified those locations that shared activity characteristics across overt and covert speech. Specifically, we asked subjects to repeat continuous sentences aloud or silently while we recorded electrical signals directly from the surface of the brain (electrocorticography (ECoG)). We then determined the relationship between cortical activity and speech output across different areas of cortex and at sub-second timescales. The results highlight a spatio-temporal progression of cortical involvement in the continuous speech process that initiates utterances in frontal-motor areas and ends with the monitoring of auditory feedback in superior temporal gyrus. Direct comparison of cortical activity related to overt versus covert conditions revealed a common network of brain regions involved in speech that may implement orthographic and phonological processing. Our results provide one of the first characterizations of the spatiotemporal electrophysiological representations of the continuous speech process, and also highlight the common neural substrate of overt and covert speech. These results thereby contribute to a refined understanding of speech functions in the human brain.

## Introduction

Attempts to describe the neurological mechanisms of speech has driven the field of neuroscience since its beginning. Some of the first neurophysiological evaluations involved stimulating brain regions related to speech and movement [1–3] and association of particular speech or language deficits with cortical lesions [4]. Since then, many studies have built upon these foundational techniques to probe the neural basis of speech function using modern imaging techniques, including functional magnetic resonance imaging (fMRI), positron emission tomography (PET), and electro- or magneto-encephalography (EEG or MEG, respectively). Using these neuroimaging and electrophysiological tools, researchers have undertaken extensive investigations to uncover the spatial or temporal properties of speech neurological processing. Several researchers have used resulting evidence to posit functional models of the speech process, and to thereby attempt to describe the nature of speech-related neural dynamics and their functional significance [5–8]. All of these models implicate the following network of cortical regions: 1) ventral (orofacial) primary motor cortex (vMC); 2) ventral pre-motor cortex (vPMC); 3) inferior frontal gyrus (IFG; loosely taken as Broca’s area); 4) somatosensory cortex; 5) primary auditory cortex (Heschl’s gyrus) and 6) peri-sylvian auditory regions (e.g., superior temporal gyrus, STG). According to most models, these regions mediate at least two major functions: 1) planning & control of the motor production of speech (articulation); and 2) perception & maintenance of hearing oneself or others speak.

At the same time, previous research has made little progress in simultaneously identifying the spatial and temporal dynamics of brain activity relating to speech, in particular for continuous speech. As a consequence, computational and theoretical models of speech function, though the result of sophisticated reasoning and experimental data, are currently based on the collective interpretation of relatively indirect evidence. For instance, studies using fMRI to examine speech production and perception have resulted in spatially precise topographies of their underlying neural correlates (see [9] for an extensive review). However, the hemodynamic response on which the blood oxygen dependent (BOLD) signal of fMRI depends, peaks approximately 4-6 seconds after the presentation of an experimental stimulus, or in the case of speech production, after the start of motor command execution. As a result, it is often necessary to design experiments around one aspect of speaking (e.g., planning, production or perception), and even then fMRI can not be used to examine within-word temporal dynamics. In contrast, EEG and MEG are well suited to describing the millisecond temporal dynamics of neurological processes and have been instrumental in our current understanding of the cognitive, linguistic and perceptual neurological mechanisms of speech. Unfortunately, both of these methods are susceptible to electromagnetic artifacts from the muscular activation of orofacial muscles used for speech production, which limits studies of speech production to mostly examining pre-vocalization processes [10, 11], or covert speech production [12] with limited investigations during overt production [13–16], though recent computational advances in blind source separation are making it more feasible to investigate overt speech production using EEG and MEG [17]. In summary, these limitations make it difficult to simultaneously observe the combined spatial and temporal dynamics during the production of words, in particular for continuously varying speech found in the production of sentences. Thus, it is logical, desirable and eventually necessary to begin to acquire data that accurately define the simultaneous spatial and temporal dynamics underlying the speech process to eventually allow us to validate and extend the existing understanding of speech and language function.

Such definition first and foremost requires an imaging technique that provides both high topographical as well as high temporal resolution of neural population-level activity. In contrast to traditional imaging techniques, intracranial electrocorticography (ECoG) has such attractive characteristics. Thus, in principle, it should capture the neural dynamics of speech quite well. Also, ECoG is relatively unaffected by artifacts [18], such as motion or myoelectrical artifacts associated with speaking. As a result, there has been a substantial increase in the number of neurophysiological studies involving the recording of ECoG to determine the neural correlates of speech and language [19]. Many of these studies cite the combined high spatial and temporal resolution of ECoG over EEG, MEG and fMRI as a primary advantage for uncovering the spatiotemporal dynamics of speech, language and hearing. Our study contributes to this line of research. In prior studies of speech production, or expressive language, subjects with implanted ECoG grids have typically been asked to perform one of three tasks: 1) Verb Generation [20, 21]; 2) Picture Naming [20, 21]; or word / syllable / phoneme repetition [21–32]. Three other recent studies investigated more complex linguistic processing including a memory recall and production task [22], morphological transformations [33] and issuing one-word answers to simple questions [34]. These studies established important characteristics of neural signatures associated with language production, but they have done so in relatively unnatural settings (such as production of individual syllables in cued experiments [35]). When more natural speech production has been available (e.g., spontaneous conversation [22, 36]), the data were analyzed across whole epochs rather than at levels suitable for spatiotemporal descriptions of speech production.

Of particular interest is the role and interaction between classically sensory or perceptual processes (e.g., auditory processing) and motor or productive processes (e.g., motor planning and execution). Additionally, and perhaps most interestingly, these recent studies all tend to provide conflicting evidence for the sequencing and importance of each underlying neural correlate of speech. The study by Edwards and colleagues [20] was foundational for demonstrating the usefulness of ECoG to the investigation of the fast-paced, and multifaceted processes underlying speech perception and production in the same experimental protocol (cf. auditory perception alone [37]). In a series of verb generation and picture naming tasks, they found that the area Spt (temporal plane) was involved in the preparation of upcoming speech vocalizations, while classically expressive areas (inferior frontal gyrus, premotor cortex) were silent. Subsequent studies [26–28] confirmed a more general result of STG activations during covert speech production tasks, which support the notion that auditory processes are the primary factor in preparing upcoming speech productions. However, several other studies [21, 22, 25, 34] came to conflicting conclusions in which only the inferior frontal and pre-central cortices are involved in speech preparation with little-to-no involvement from auditory regions.

It is possible that these conflicting results can be resolved by using more realistic speech production experimental tasks in which participants generate continuous and fluent speech. In contrast, many of the prior studies required study participant responses that consists of just a single word or syllable (with the exception of [31]), which we believe is not indicative of continuously varying, fluent speech. Hence, a characterization of the neural signatures relating to continuous, and thus more natural, overt and covert speech does not yet exist, but is important for refining our understanding of the mechanisms underlying fluent verbal communication.

In the present study, we were motivated to reconcile these seemingly mutually exclusive interpretations. To direct our exploration of speech production, we first examine the methods used in these previous studies to determine a trajectory of investigation. Each of the previous studies involves production of a word or syllable in isolation, but vary on the eliciting cue (e.g., visual words, auditory stimulus or question / response). The studies all define analysis intervals for examining pre-response versus post-response neural activity, but vary in the time windows used to define those intervals. In those studies that utilize a covert, or imagined speech task, the interval from the overt condition counterpart is used for data analysis. Last, each study also varies in the length of time between presentation of the visual or auditory stimulus and the expected response period. Therefore, we employed a paradigm that elicited fluent, continuous speech production, using familiar stimuli for speech repetition and analyzed the resulting neurological activity relative to the observed speech acoustics. These factors will allow interpretation of neurological activity that is similar to what is expected in normal conversation with time alignment to the main behavioral outcome of speech production (i.e., the acoustic signal). We also employ a covert condition for comparison of neurological activity during imagined speech production to actual speech production. Our use of sentence stimuli is a direct response to comments by Kunii and colleagues [21] who commented that their results were somewhat incomplete despite using a unique speech elicitation protocol because they were not able to comment on more complex forms of speech production such as sentences.

In our approach, we examine the spatio-temporal dynamics of population-level ECoG activity recorded from the left hemisphere during long-duration (i.e., paragraph-length) continuous overt and covert speech production in order to obtain relationships between fluent speaking and its underlying neurological processes. In order to make comparisons with prior word repetition studies of overt and covert speech production (e.g., [13, 20, 28, 38]), we utilized visual presentation of the stimulus words on a computer screen that were continuously updated reflecting the subjects’ current position in the larger stimulus paragraph. We obtained ECoG activity from eight human subjects who participated in this continuous word repetition task. During overt production, we hypothesized that ECoG activations observed prior to their corresponding acoustic output would be located primarily in frontal cortex (specifically vMC, vPMC and IFG) and likely relate to planning and execution of upcoming utterances, whereas ECoG activations that follow acoustic output would be observed primarily in temporal locations (e.g., STG) and likely relate to hearing oneself and continued online maintenance of production. In addition, prior work suggested that temporal regions may contribute to the neural activity immediately prior to the auditory feedback signal by contributing to speech planning [8] and phonological processing [39, 40]. However, these roles typically cannot be disambiguated from primary auditory processing since continuous speaking involves simultaneous and overlapping motor production and sensory perception. Therefore, we employ a covert speaking paradigm to isolate those neurological processes used for planning and execution of speech.

Our overt condition results show the entire speech network (frontal and temporal locations) were active at the moment of speech production indicated by neural activity coincident with acoustic output. The combined spatio-temporal analysis clarified the sequencing of these areas activity, though by focusing on specific neural regions of interest we were required to limit the specificity of the speech features. In contrast, another study from our group focused on speech features (e.g., the place and manner of speech) while limiting the analysis of neural region of interest [41]. In the present study, our results showed that fronto-motor locations became active prior to auditory regions, indicating their role in the planning and execution of speech, while auditory regions were primarily active for the role of acoustic feedback processing. Similar analyses for the covert condition, in which we related ECoG readings to the actual acoustic output obtained from the overt condition, did not reveal any significant relationship. Thus, ECoG signals during covert speech production do not appear to be encoding the acoustics of the intended speech. In marked contrast, the direct comparison of the overt and covert conditions revealed additional areas of common neurological activity, which are likely related to oculomotor control, verbal working memory and auditory processing that are common to both tasks.

## Material and Methods

### Human subjects

The eight subjects who participated in this study were patients with intractable epilepsy at Albany Medical Center. Subjects underwent temporary placement of subdural electrode arrays to localize seizure foci prior to surgical resection of epileptic tissue. All gave informed consent to participate in the study, which was approved by the Institutional Review Boards of the hospital. The subjects had performance IQs of at least 85 and were mentally, visually and physically capable of performing the task. Assessments of language lateralization were not available in our subjects. Hence, we do not draw any conclusions related to the laterality of specific aspects of language function in our study.

The implanted electrode grids (Ad-Tech Medical Corp., Racine, WI) consisted of platinum-iridium electrodes (4 mm in diameter, 2.3 mm exposed) that were embedded in silicone and spaced at an inter-electrode distance of 1 cm. One subject (H) was implanted with an electrode grid (PMT Corp., Chanhassen, MN) with 6 mm inter-electrode spacing. Grid placement and duration of ECoG monitoring were based solely on the requirements of the clinical evaluation without any consideration of this study. A summary of the participants’ clinical profile, implanted electrodes, and task-related information is given in Table 1.

**Table 1:** Clinical profiles of all study participants and characteristics of collected data. Handedness, performance IQ (PIQ) and verbal IQ (VIQ) were assessed prior to participation. The characteristics of the word stimuli are shown in the final three columns (Length: number of words in the passage; Duration: length of time needed to produce the passage in the overt condition; Speed: display rate of word stimuli). All participants were right-handed with seizure foci on the left hemisphere. All electrode grids were implanted on the left hemisphere and the number of electrodes reported include only those that were not excluded due to artifacts. The number and placement of electrodes were determined according to clinical needs with no consideration for this study.

Subj.	Age	Sex	PIQ	VIQ	Seizure Focus	Num. Elec.	Length (words)	Duration (secs)	Speed (% of screen width / sec)
A	29	F	136	118	Temporal	96	278	279.87	30
B	30	M	90	64	Temporal	82	109	129.87	25
C	29	F	90	91	Temporal	101	283	310.93	25
D	19	M	85	87	Frontal	75	411	470.90	25
E	26	F	117	106	Temporal	67	411	341.77	35
F	56	M	87	82	Temporal	94	411	465.67	25
G	45	M	105	93	Temporal	56	278	262.30	30
H	29	F	95	111	Temporal	120	411	590.10	20

**Fig 1:**
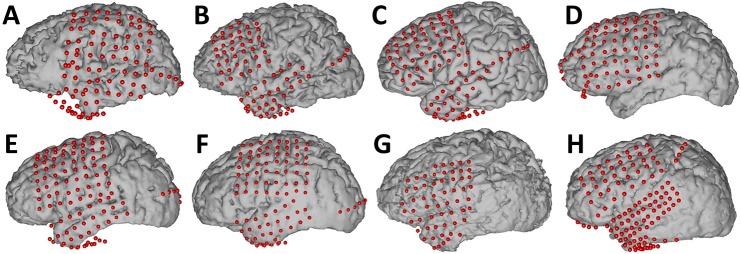
Placement of electrodes on all 8 subjects. Electrode locations were identified in a post-operative CT, co-registered to preoperative MRI, and transformed into a joint (Talairach) space for comparison across subjects. An average brain is shown for Subject C as a 3D rendering was not available.

Each subject had postoperative anterior-posterior and lateral radiographs, as well as computer tomography (CT) scans to verify grid locations. Three-dimensional cortical models of individual subjects were generated using pre-operative structural magnetic resonance (MR) imaging. These MR images were co-registered with the post-operative CT images using Curry software (Compumedics, Charlotte, NC) to identify electrode locations (see Fig 1). We derived cortical locations in a common space using Talairach’s Co-Planar Stereotaxic Atlas of the Human Brain [42] and a Talairach transformation (http://www.talairach.org). Cortical activation maps were generated using the NeuralAct package [43]. Activation maps computed across subjects were projected on the three-dimensional cortical template provided by the Montreal Neurological Institute (MNI) (http://www.bic.mni.mcgill.ca).

### Data collection

**Fig 2:**
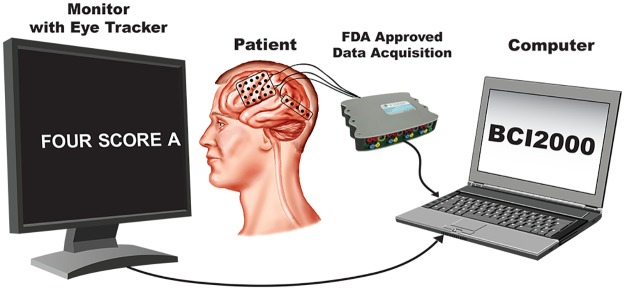
Diagram of the experimental setup. The subject was presented with scrolling text on a computer screen with built-in eye tracker, which verified the location of eye gaze on the screen during data acquisition. The eye tracker and data acquisition devices were interfaced with a computer running BCI2000.

The experimental setup is depicted in Fig 2. ECoG signals were recorded at the bedside using eight 16-channel g.USBamp biosignal acquisition devices (g.tec, Graz, Austria). ECoG signals and the signal from a microphone were simultaneously digitized at a sampling rate of 9600 Hz. Electrode contacts distant from epileptic foci and areas of interest were used for reference and ground. Recordings were visually inspected offline for environmental artifacts and inter-ictal activity. Channels that did not contain ECoG activity, e.g., due to broken wires, were removed from the analysis, which left between 56-120 channels each for subjects A-H.

In addition to recording brain activity, the subjects’ eye gaze was recorded using a monitor with a built-in eye tracking system (Tobii Tech., Stockholm, Sweden). The eye tracker was calibrated to each subject at the beginning of the experimental session using custom software. Data collection from the biosignal acquisition devices, microphone, eye tracker, as well as control of the experimental paradigm were accomplished simultaneously using BCI2000 software [44, 45].

## Experimental paradigm

During the study, each subject was seated in a semi-recumbent position in a hospital bed about 1 m from a video screen. During the experiment, the text of a famous passage (e.g., Gettysburg Address or Humpty Dumpty nursery rhyme) ranging from 109 to 411 words, scrolled from right to left across the video screen at a constant rate between 20% and 35% per second chosen based on the preference and cognitive capabilities of each subject, and resulted in run durations between 129.9 and 590.1 seconds (details in Table 1). For each subject, this rate was selected to be comfortable based on the subject’s attentiveness and cognitive/verbal ability. For all subjects, we recorded ECoG in two different conditions in separate experimental runs. In one condition, the subject was instructed to repeat the presented text out loud, while in the other to silently imagine producing the presented text (as opposed to imagine hearing the text), i.e., “overt” and “covert” tasks, respectively. The distinction between imagined productions versus imagined hearing is important, as previous studies have indicated differential neurological processing may be involved for each of those tasks [46]. Further, during the covert task, subjects were additionally instructed to avoid making any head or mouth movements and to remain completely silent, without any vocalization or subvocalization (similar to instructions in prior covert production studies [46, 47]). Each overt run was followed by a covert run with the identical scrolling text and scrolling speed. We additionally chose these familiar stimuli to ensure some amount of predictability that would reduce the cognitive effort needed for subjects to perform the task and allow us to focus on the processes underlying covert speaking. It is possible that participants may have anticipated some of the stimuli due to prior knowledge or task learning (i.e., covert always following overt), however, the effect on our study would be to reduce the calculated relationship between overt and covert speech processing. Viewed that way, our results are a conservative estimate of the correlations between the brain responses involved in overt and covert speech.

### Data analysis

The raw ECoG signal from each electrode was first high-pass filtered with a cutoff of 0.01 Hz to remove low-frequency trends, and then spatially re-referenced using a common average reference (CAR) montage. The resulting signals were low-pass filtered and decimated to 400 Hz. A finite-impulse-response (FIR) notch filter in the 116–124 Hz range with zero-phase (forward and inverse) and -60 dB stop-band attenuation was used to remove the 120 Hz line noise harmonic. A separate bandpass filter in the range 70–170 Hz with zero-phase and -60 dB stop-band attenuation was applied to the notch-filtered signals to extract the broadband ECoG activity.

**Fig 3:**
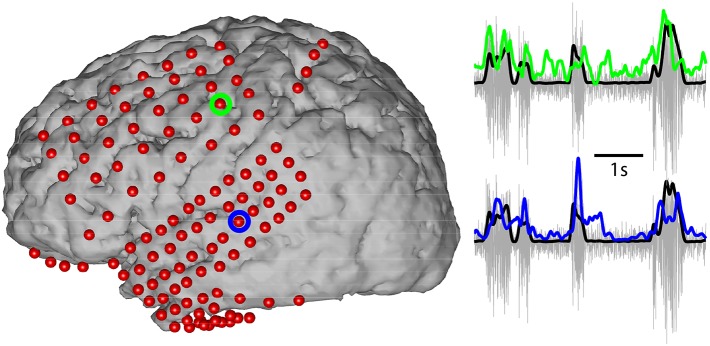
Exemplary time courses (right) of broadband power modulations during vocalizations are shown for two exemplary electrodes over motor (green) and auditory (blue) cortex for one subject (H). The locations of each ECoG electrode are shown as red dots, while green and blue circled locations correspond to precentral and superior temporal gyrus electrodes whose time courses are shown on the right. The vocalizations were synchronously recorded with ECoG signals via a microphone and are shown in gray with the rectified amplitude envelope in black.

We then computed the envelope for both the broadband activity and the microphone speech intensity as the magnitude of the analytic signal obtained from the Hilbert transform [48]. The speech intensity envelope was chosen as a proxy for speech production that is globally related to the respiratory, phonatory and articulatory processes during speech, and prior results have shown it is well represented by ECoG broadband power [49]. For instance, in a simple sense, the intensity envelope is minimal during silences (no respiration, no phonation) and maximal during speech. It does, however, have a more complex relationship to speech acoustics and articulation as it increases with first formant frequency or with the proximity of the first two formants (the formants themselves are related to movements of the speech articulators for altering the resonance of the oral cavity) [50]. The broadband (70–170 Hz) power was then smoothed using an eighth-order lowpass Chebyshev Type I filter with cutoff frequency of 8 Hz and decimated to 100 Hz, similar to methods used for analyzing auditory processing using ECoG [51]. The smoothed speech envelope is used here as a reasonable feature for relating neural activity to macro-level behavior during speech production. Additionally, the Hilbert transform is widely utilized in neuroscience, especially for ECoG studies when computing the magnitude of the relatively wide 70–170 Hz band [52, 53]. In control analyses, we produced the main results of this study also using a simple band-power estimate (squaring followed by low-pass filtering) and compared them to the results that we derived using the Hilbert transform. The results were practically identical. An example of both the ECoG broadband envelope and speech intensity envelope is given in Fig 3 for two electrodes with demonstrable correlation between the neural and speech signals.

To generate the main results of this study, we related the time course of ECoG broadband power (e.g., Fig 3) at a particular location to the envelope of speech intensity. The whole speech intensity envelope was correlated to the ECoG broadband power in contrast to alternative procedures that utilize event-based windows averaged over trials. To do this, we first calculated Pearson’s correlation coefficient between the time courses of broadband power and speech intensity at varying temporal offsets (-500 ms and +500 ms, equally spaced by 20 ms). Correlation analysis has been previously shown to be a faithful representation of neural activity related to a specific task, including processing of speech [29], specifically the speech intensity envelope [49]. This analysis is similar to performing a cross-correlation between the two signals in Fig 3. The statistical significance of the resulting correlation coefficients was computed using a bootstrapping randomization test, in which the broadband power samples for each channel were randomly scrambled across time to preserve their statistical properties, but destroy temporal relationships. We duplicated this analysis using time-reversed bootstrapping randomization test (as opposed to time-scrambled) to determine whether the autocorrelation structure of the underlying signal had any effects on the statistical correlations at the heart of our analysis. The results were virtually identical, which suggests that the autocorrelation of our data have only negligible effects on our results. The cross-correlation procedure described above was repeated on 1,000 scrambled data sets. The resulting correlation coefficients were fit to a beta distribution; the p-value of the original (unscrambled) data was computed based on this distribution and Bonferroni-corrected for the total number of electrodes pooled across subjects (N = 691 electrodes). The beta distribution was chosen based on an analysis that indicated its effectiveness in modeling correlation coefficient random samples, especially accounting for the tails. A Kolomogorov-Smirnov test confirmed (p = 0.05) the statistical equivalence of the scrambled and beta distributions. Finally, we quantified that relationship by computing the negative logarithm of the corrected p-values, which resulted in one “activation index” for each electrode. The negative logarithm has been used previously to represent similar correlation analyses [51, 54–58], and our results were qualitatively similar to those based on the correlation coefficients themselves indicating that either feature is acceptable for our purposes., and has been referred to as the activation index [41, 59], tuning index [54], significance index [56, 57, 60], confidence index [55, 61], or similar terms. We used the number of electrodes with statistically significant correlations (-log p>9.53, after correction for 691 electrodes at p = 0.05), as a measure of neural activity during speech processing, which yielded 3–27 significant electrodes per subject and 114 total electrodes active in at least one time bin over all subjects. We then used a binomial test at each time point to determine the intervals in which there was greater involvement of either fronto-motor or temporal regions.

We next estimated the speech output activation indices for the covert condition. The envelope of the speech signal can readily be calculated for the overt condition, but not the covert condition, which precludes its direct use as a behavioral correlate. As an alternative, we utilized the speech intensity envelope of the overt condition as a proxy for the intended acoustic speech output. We also used identical visual stimulus scrolling speed in both the overt and covert conditions to help maintain timing between conditions. This analysis did not lead to statistically significant results limiting our ability to describe the relationship of covert speech to intended respiration, phonation and articulation (only one electrode from each subject was statistically significantly correlated with the overt speech intensity envelope). Instead, we compared broadband power during the covert speech condition to the ECoG broadband power observed in the overt condition. In this direct comparison, we computed activation indices based on the zero-lag correlation of the overt condition ECoG broadband power to that of the covert condition (e.g., assuming each production attempt occurred with similar timing). In a preliminary analysis we also computed activation indices at all lags (-500 ms to +500 ms) as well as using an “optimal-lag” in which dynamic time warping was used to match the covert condition ECoG broadband power to that of the overt condition. Analysis of the comparison at all lags revealed no substantial relationships between the two conditions beyond the zero-lag comparison. In the optimal-lag analysis, we compared the differences between the warped and unwarped correlations and found similar results with no statistically significant differences. We therefore proceeded with the zero-lag comparison for simplicity, with the assurance that other lag offsets were not expected to provide significantly different results.

## Results

### ECoG responses to continuous overt speech

**Fig 4:**
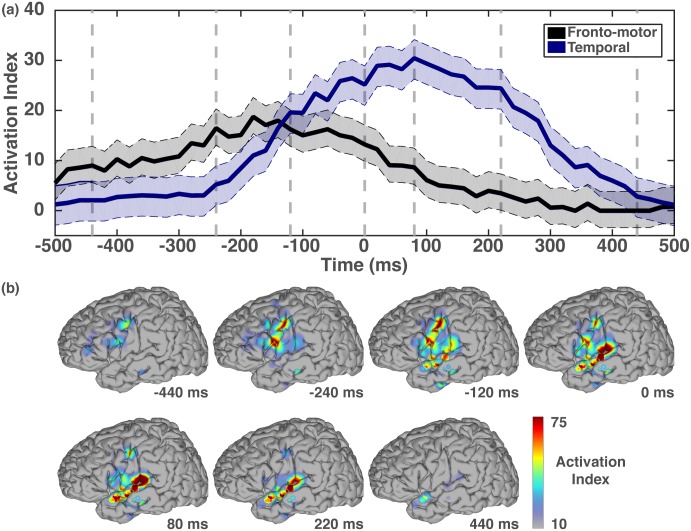
**(a) The average activation indices for electrodes with statistically significant correlations for two regions of interest (ROI) are illustrated.** The two ROIs are areas in fronto-motor cortex (Broca’s area, ventral premotor cortex [Premotor] and primary motor cortex [M1]), shown in black, and areas in temporal cortex (middle and posterior superior temporal gyrus [mSTG, pSTG] and middle temporal gyrus [MTG]), shown in blue. The shaded boundaries indicate the 95% confidence intervals of the average activation indices. Dashed grey lines identify time points of interest including a baseline (-440 ms), rising fronto-motor response (-240 ms), equal frontal-motor and auditory response (-120 ms), zero-lag correlation (0 ms), peak auditory response (80 ms), falling auditory response (220 ms) and post-speech baseline (440 ms). **(b) The spatiotemporal topography of statistically significant correlations of broadband power with speech intensity across the entire overt speech production task are shown at the time points of interest identified in (a).** Color values represent activation indices (see color bar). The topographies identify the same general areas (motor, pre-motor and auditory regions) identified previously using functional imaging studies of the speech motor network, but for the first time provide direct observation of their temporal evolution from motor to sensory areas.

**Fig 5:**
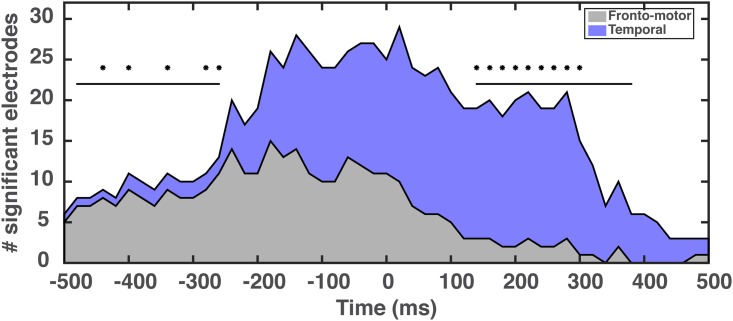
The number of electrodes with statistically significant correlations to the speech envelope in the overt condition grouped according to major area: fronto-motor (including IFG, premotor and primary motor cortex) and temporal (including STG and MTG). The parietal area (SMG) was minimally correlated to the speech envelope and is not included in this figure and analysis. Statistical tests (binominal test with FDR correction at each time point, p_fdr_ = 0.05) determined that fronto-motor areas has statistically more activated electrodes than temporal areas at the -440, -400, -340, -280 and -260 ms time points. Similar analyses found that temporal areas had statistically more activated electrodes than fronto-motor regions at the 140–300 ms time points. (The black lines indicate the time intervals for which the binomial test was significant at the p_fdr_ = 0.06 level).

**Fig 6:**
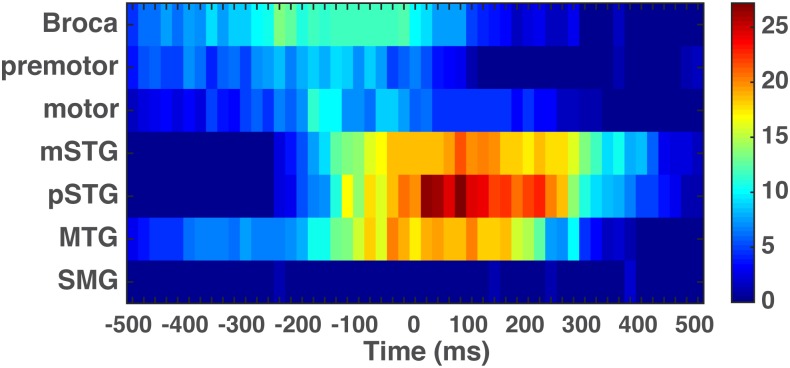
The average activation indices for electrodes with statistically significant correlations by ROI described in Fig 4. The average activation index is represented by a color scale, with more blue colors indicating less statistically significant correlation of ECoG band power to speech output and more red colors corresponding to greater statistical significance of the correlation.

The primary results of this study are given in Figs 4–6 and in S1 Video. Fig 4 shows the average activation indices for the two major functional regions (fronto-motor and temporal) (top) and the spatio-temporal progression of neural activation indices for all eight subjects combined on an average cortical template (bottom). Across all subjects, 114 of 691 unique electrodes reported statistically significant correlation to the speech intensity signal for at least one time bin, with a maximum of 39 electrodes in any one bin.

We further tested the hypothesis that fronto-motor regions are preferentially involved prior to speech output, and temporal are preferentially involved concurrent with or after speech output (Fig 5). To do this, we established the number of electrodes with statistically significant correlations to the speech envelope at each time bin, and then, for each bin, used a binomial test to determine whether the number of significant electrodes was different in the fronto-motor and temporal regions. The resulting p-values were compared using the false discovery rate (FDR). The results, shown in Fig 5, demonstrate a statistically significant difference in the occurrences of fronto-motor electrodes relative to the temporal electrodes at -440, -400, -340, -280 and -260 ms (p_fdr_ = 0.05) and the temporal electrodes relative to the fronto-motor electrodes in the interval 140–300 ms (p_fdr_ = 0.05). Relaxing the significance threshold to (p_fdr_ = 0.06) further reveals that the fronto-motor electrodes occur more frequently in the range of -480 – -260 ms and the temporal electrodes in the range 120–380 ms. Electrodes in the two regions occur equally frequently in the range of -240–120 ms. Fig 6 provides a qualitative graphical description of the spatio-temporal progression of the seven major brain areas involved in the fronto-motor and temporal region analyses. This image conveys the involvement of the ventral precentral gyrus (serving both premotor and primary motor functions) and the inferior frontal gyrus (e.g., Broca’s area) at negative lags that diminishes in magnitude at positive lags. In marked contrast, activation indices in auditory areas (middle temporal gyrus & middle and posterior superior temporal gyrus) are predominantly observed at positive lags.

### Shared ECoG responses for overt and covert conditions

**Fig 7:**
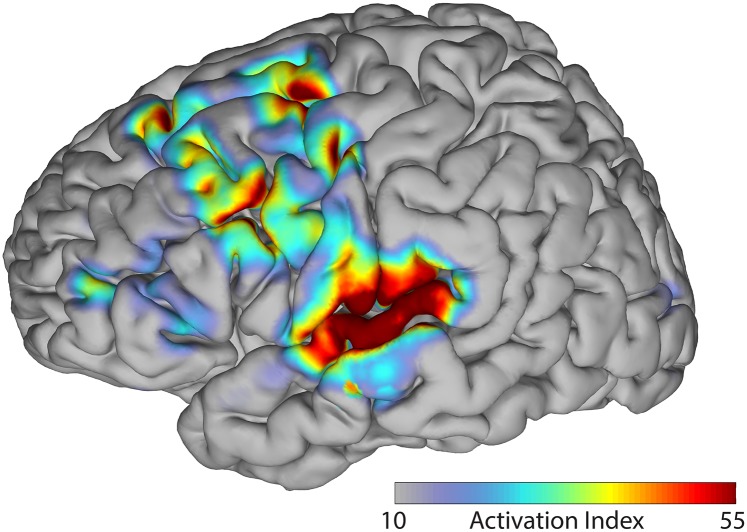
The spatial topography of statistically significant relationships between brain signals in the overt and covert condition over the entire continuous session. Color values represent values of activation index (see color bar). Strong auditory cortex involvement is observed in the superior temporal gyrus, along with lesser activation along a path from the DLPFC, FEF, premotor and motor regions; IFG and vMC sites found in the overt and covert conditions alone are notably absent.

We then identified those locations whose ECoG activity was similar across overt and covert conditions. The results of this analysis revealed that 71 of 691 electrodes had activation indices that were statistically larger than those expected due to chance alone (see Fig 7). These electrodes were located primarily in auditory cortex (STG), premotor and primary motor cortex, frontal eye fields (FEF) and dorsolateral prefrontal cortex (DLPFC). Thus, the spatial topography of these results define those cortical regions whose activity is common to both the overt and covert conditions in our task, independent from any acoustic signal (e.g., speech intensity). The specific motor cortical responses previously observed in the overt condition are notably absent in these combined results (other areas of motor cortex are active) while statistically significant STG responses are present in both analyses. Taken together, these results suggest three implications: 1) speech intensity, an acoustic measure, is an appropriate representation for overt speech production (which includes auditory feedback and is hypothesized to employ auditory information for planning speech [5]), but not for covert speech; 2) speech intensity is well suited to describe auditory cortical processing areas; and 3) the brain regions common to overt and covert speech are not related to the speech intensity envelope. We further elaborate on these implications in the Discussion below.

## Discussion

### General comments

In this paper, we provide an account of the spatio-temporal evolution of electrophysiological activation involved in production of continuously varying overt and covert speech—from planning, to execution and sensory feedback maintenance. Our use of the continuous speaking task allowed us to extend previous results that primarily focused on simple utterances (e.g., syllables, words and phrases) toward more realistic speech actions involving long sequences of sentence-length utterances found in typical conversation. The intent of this type of experimental paradigm is to facilitate natural and fluent speech in contrast to experimentally controlled single utterances. Our sentence and paragraph-length stimuli were designed with continuous fluency in mind, as in the conversational tasks in [22, 36]. In these studies, the subjects’ continuous speech was parsed into one-word epochs and subsequently analyzed to determine the differences in neural activity as a result of speaking and listening. In our study, we extend these results by using continuous measures to analyze fluent speech. That is, we do not epoch the speech and ECoG data according to individually produced words, syllables or phonemes. Rather, we directly compare the speech acoustics (namely the speech intensity envelope) with ECoG broadband power over the entire stimulus. This is notable, because we obtain results that are consistent with the overt speech production literature generally (and these two studies specifically) without the need for separate parcellation, which is time-consuming and error-prone. Our continuous task also allows for more accurate analysis of the covert condition; we directly compared the full duration of neurological activity in the overt and covert tasks, while prior studies of covert speech using a word-based epoch analysis, which must estimate the onset times each covertly spoken word.

The following details are helpful for interpreting our results. First, all ECoG recordings are obtained by placing electrode grids directly on the cortical surface. Thus, electrodes may not be placed precisely on individual gyri, or over specific sulci. The locations in Figs 4 and 7 both result from co-registration of pre-operative MR images and post-operative CT images. The error of such co-registration procedures have been reported to be in the range of 5-6 mm [62]. They were then transformed into Talairach coordinates. Second, for our calculation of activation indices, we used the envelope of the speech output as a marker of speech production, which is an appropriate, but certainly not the only possible indicator of speech function (cf. pitch, articulatory kinematics or muscular activity).

The discussion in the following paragraphs examines direct effects of overt and covert speaking on spatiotemporal cortical activation indices. We first consider the contributions of neural processing to overt speech production and offer interpretations in the context of contemporary views of both speech production and perception. We follow this discussion with examination of the covert task results and their relationship to overt speech production. We finish with a discussion of the implications of our results on future ECoG study of speech production.

### Overt condition

This study provides a spatiotemporal description of the cortical areas involved in overt speech production using electrocorticography during continuous speech. The regions found here have been previously hypothesized to activate in a coordinated manner [5, 7, 8, 35], but direct electrophysiological confirmation of the resulting expectations have been lacking.

Our main result was the finding of a network of fronto-motor and temporal regions that are preferentially activated during overt speech production when compared against auditory speech output. This network included primary motor and premotor cortex, Broca’s area, and peri-sylvian auditory areas (see Figs 4 and 6 and S1 Video). Fronto-motor regions (vMC, vPMC and IFG) are thought to contribute most to planning and execution of speech utterances, while temporal-parietal region activations represent sensory feedback control mechanisms, including middle and posterior superior temporal gyrus as well as lateral areas overlying the temporal plane, a region often cited as critical to the acoustic perception of speech [7, 8, 63].

In addition to topographical analyses, our results provide an account of the temporal progression of neural activations as they modulate during speech production. Earlier neuroimaging results and theoretical models suggest a specific temporal organization of neural activations that represent planning, execution and feedback control mechanisms. In our study, we were able to effectively sample from each of these processes owing to the constant updating of plans and productions needed for a continuously varying word repetition speech task. In this task, speakers also experience constant auditory feedback of their own productions, which presumably elicits monitoring and control processes.

From Fig 5, we observe neural involvement that begins with frontal-motor activations preceding resultant speech output, and putative vocal muscular excitation, which then shifts toward sensory feedback processing in primary auditory regions. Inspection of the time courses and topography of these activations suggests that they originate in inferior frontal gyrus and ventral precentral gyrus (from Fig 4); strong auditory cortex activations are present as early as -240 ms relative to speech output (see Figs 4 and 5). In this study, we utilized the vocalized speech intensity as a proxy for speech output; however, prior studies have shown that muscular activation precedes observation of the speech acoustical signal by 100 to 200 ms on average [64] but up to 500 ms in the extreme [65]. Therefore, an approximation of the cross-correlation to the actual speech motor activation can be estimated by adding this offset (100–200 ms) to the cross-correlation latencies. Accordingly, the auditory cortex activation indices would be present near 0 ms (motor command corrected).

Furthermore, auditory (temporal) cortex activations tend to follow frontal-motor activations, and are not observed during expected motor preparation time intervals (cf. [20]). This pattern is in conflict with predictions by auditory-based theories of speech initiation [8, 35], but supports other frontal-motor-based theories [5, 63] and is consistent with other previous ECoG studies of speech production [21, 22, 25, 34]. Importantly, our study derives from a word repetition task, which is just one of many lead-in processes [35] for speech production, examples of which include picture naming, verb generation, pseudoword reading and auditory word repetition. Prior work involving the overt or covert repetition of acoustically presented syllables and words typically involve auditory processing areas prior to frontal-motor regions [20, 25, 26]. It remains to be discovered whether auditory-based or frontal-motor-based networks mediate continuous speech production following each of these lead-in processes.

In addition to addressing issues in speech production, our results for the overt condition provide important insights into speech sensory processes. Specifically, speech processing is often thought of as two distinct, but interconnected phases: receptive and expressive. One recent receptive speech study [66] examined the neurological processes active during the acoustic perception of proper nouns and verbs. The authors report primarily on an observed sequence of temporal lobe activations following the presentation of the auditory stimulus beginning with posterior STG and followed by middle STG and the superior temporal sulcus. Also present are premotor cortical activations that begin around the same time as the earliest auditory cortical responses. Though the authors do not offer any interpretations of these coincident activations, the presence of both modalities is an important observation of the interactions between receptive and expressive speech. In another study by Mainey et al. [38], speech processes were studied in a verbal working memory paradigm. In this example, a visual stimulus was presented along with a separate cue that indicated whether a subject should remember or ignore the target stimulus. At the end of an experimental run, the target stimuli marked for encoding were verbally repeated. Here, the authors observe activations in inferior frontal gyrus (Broca’s area) prior to auditory cortical responses, suggesting an important role in the phonological loop thought to facilitate verbal working memory. Premotor and motor cortical responses were also observed in the verbal response period. The data presented in the present study are focused primarily on expressive elements of speech, i.e., the planning, execution and online maintenance (through feedback) of uttered sounds rather than comprehension of incoming acoustic signals, or receptive speech processing. Nonetheless, our data do provide observations of receptive processing during the act of speaking, which is widely believed to be an auditory monitor of self vocalizations [22, 39, 53]. Our results for the overt condition demonstrate activations in auditory cortex coincident or following resultant vocalization, which corroborates prior findings by Crone and colleagues [39] and are supported by more recent findings regarding speech articulation [67, 68].

The auditory cortical contributions found in our overt condition analysis at negative lags may alternatively interpreted as an intermediate phonological processing stage involved in word reading tasks [39]. The temporal envelope of this functional processing stage is reported to span from 200 to 600 ms after presentation of a visual word stimulus, ending approximately 120 ms prior to speech onset, with a second temporal envelope of auditory activity during vocalized responses [39]. Similarly, in our analysis, the auditory response also begins prior to the current speech output; however, it starts just 240 ms prior in contrast to 500 ms in the earlier study. There is also no morphological difference in the temporal envelopes in the current analysis between early and late latency auditory responses, which is in contrast to the two separable patterns of activity observed in the earlier study [39]. It is entirely possible that these differences can be attributed to the online nature of our continuous sight reading task, which did not permit prolonged attention to any one visual word representation as they scrolled across the screen. The presence of distractors and the fluidity of the task may have altered the phonological processing response to facilitate the on-demand requirements of our speech production task.

### Covert condition and shared overt-covert response

Our analysis of the data for the covert condition was designed to identify those components of the speech network that are involved in the planning and execution of speech motor programs without the confound of simultaneous auditory processing of self vocalizations present in the overt condition. When comparing ECoG signals from the covert condition to the overt condition speech intensity envelope, we found that the observed neural signals are only minimally related to the temporal envelope of (the proxy) speech output. We conclude that this comparison is not a reliable description of the covert speaking process and that future research should focus on investigating alternative features that better represent covert speech processing. In contrast, the overt versus covert analysis is not affected by choice of speech representation, as the spatiotemporal ECoG signals are directly compared between the two conditions. The results from this analysis implicates frontal-motor and auditory areas in the core task of continuous sight reading with mental preparation of speech output. Between the two conditions, auditory cortex is consistently activated, as are ventral premotor & primary motor areas. Slight co-activation can be observed in the inferior frontal gyrus, but not to the extent observed in the analysis of the overt condition. Further, the overt versus covert condition results highlight two additional areas, frontal eye fields (FEF) and dorsolateral prefrontal cortex (DLPFC), which perform processing functions common to both conditions.

Our results for the ventral motor and auditory responses in continuous overt and covert speech production are also corroborated by earlier studies of mono-syllabic word repetition. In [26, 28], subjects were asked to either view or listen to the word stimuli, then to produce them either overtly or covertly. The authors in [26] mention that fronto-motor areas are active during the covert speaking condition, but that the auditory contributions (specifically posterior STG) appear to be the primary response. We find a similar response asymmetry in the overt versus covert condition analysis. In both the mono-syllabic word repetition and continuous speech tasks, it is likely that auditory cortex is being continuously activated with other working memory networks (e.g., DLPFC) for storing the stimulus sound. In the study by Pei and colleagues, the entire auditory-phonological representation of the target word is stored, while in the present study, auditory cortex may be involved in storing intermediate phonological representations as the stimulus scrolls across the computer screen [26]. Again, as in [26], the co-activation of fronto-motor cortex is relatively diminished compared to the overt condition. Such reduction may result from transient motor involvement limited to a precise time for covert production, which is in contrast to constant updating of auditory and phonological representations of upcoming speech utterances.

### Relationship to word processing

Previous neuroimaging studies have indicated involvement of the left occipitotemporal, inferior frontal and posterior superior temporal cortices as subjects performed overt word reading tasks [40, 69–73]. Further evidence from Indefrey and Levelt [35] indicates that processing of written words likely occurs prior to motor planning and articulation. In the present study, we employed a task that required subjects to first read a word on the screen, then to repeat that word aloud or silently in rapid fluent succession. Therefore, our task involves certain aspects of visual word processing, including primary visual perception and semantic processing as well as speech motor preparation, production and maintenance (the main focus of our work). The electrodes used in this study were placed on the lateral surface of the brain, but did not include depth electrodes. Thus, given the data at hand, we could not discern whether any activations from inferior temporal areas originate from the lateral temporal cortex or the occipitotemporal cortex (e.g., visual word form area, left fusiform gyrus [72]). However, the inferior frontal areas do show statistically significant activation indices prior to any posterior superior temporal areas in the overt condition (beginning at -500 ms), which is consistent with both the word reading results and articulatory planning and output for speech production [71]. There are also some minimal statistically significant activation indices in the superior temporal gyrus in the overt condition beginning at -240 ms, which increase in amplitude, peaking at +80 ms and terminating at +500 ms. These early activations may also support semantic processes related to word reading [69], and provide an alternative interpretation of the inferior frontal activities in which they are an additional part of the semantic retrieval system as in [40, 70]. That said, the overwhelming fronto-motor responses near at negative lags (-500 – -200) ms occur prior to the strong activation of temporal cortex locations; as such, they are likely due to the planning and execution of speech motor plans [71] rather than as a result of temporal cortex processing of semantic information. These results combined with our analysis method of comparing neural activity to speech intensity, an acoustic measure that is related to speech motor control, suggest our study focuses on processes other than word reading, which likely occurs far earlier than 500 ms prior to initiation of speech articulation or imagery commands [35].

## Conclusions

A major implication of this work is that the choice of behavioral correlate, whether overtly or covertly produced, is critical in isolating the many overlapping processes involved in speech production. In our analysis, speech output intensity allowed us to identify the neural correlates of overt, but not covert, speech very well. In contrast, the correlation analysis of overt to covert condition ECoG activations revealed a visual-spatial phonological processing network common to both of our tasks, yet different from the primary motor and sensory network observed in the overt condition alone. Both networks are present and active during our sight-speaking task, which implies that other observable and “intended” behavioral output features may be useful for uncovering the temporal evolution of covert speech. In the case of sight reading with covert speech production, the movements of the eyes would seem an appropriate feature given FEF involvement in the common overt-covert speech processing network.

It is also important to note the potential sources of error when analyzing ECoG data. In particular, ECoG is necessarily recorded from individuals with neurological deficits (most commonly, people with epilepsy). While we excluded electrodes that did not clearly contain normal ECoG activity, we cannot exclude the possibility that these subjects had in part abnormal functional neuroanatomy. At the same time, the results derived using the ECoG method usually conform to expectations given by other imaging techniques. In addition, just as any other imaging technique, ECoG data have intersubject and intrasubject variability. Some prior ECoG studies account for this variability through single-subject analyses (e.g., [20]). In the present study, we perform population-level analyses, which produces results that may generalize to larger populations. A consequence of this population-level analysis is a tradeoff between spatiotemporal specificity (i.e., the ability to link neural activations to specific times) and behavioral specificity (i.e., speech envelope vs other acoustic and articulatory features). Furthermore, in this study, we opted to use a coarse behavioral correlate, the speech intensity envelope, as a feature that could be related to specific spatiotemporal neural activations. In a related study from our group, we chose to examine specific phonological and articulatory features of speech, but were not able to make strong claims about the temporal progression of individual brain regions [41]. Taken together, our two studies provide complementary accounts of the spatiotemporal progression of neural activity underlying speech production. Further research on the electrophysiological substrates of speech production may lead to advancements in neural speech prostheses. Finally, we acknowledge that the number of subjects in the present study is small compared to large speech-language studies using neuroimaging, but is larger than most other ECoG studies (i.e., 8 subjects in the current study vs. 3–5 in typical ECoG studies). Thus, we are careful to limit our conclusions to those that can be supported by the data. We believe this work is very exciting and holds great promise, and future studies will be able to build from our work to make additional interpretations.

## Supporting Information

S1 Video The spatiotemporal topography of ECoG broadband power to speech intensity. (MP4)
